# Rhein Inhibits NLRP3 Activation and Alleviates Microglial Pyroptosis After Intracerebral Hemorrhage in Rats

**DOI:** 10.1002/brb3.71230

**Published:** 2026-01-28

**Authors:** Adalaiti Aimaiti, Qian Li, Tao Liu, Chen Chen, Xiaolin Xie, Jianshu Chu, Dilihumaer Nuermaimaiti, Yan Hu

**Affiliations:** ^1^ Xinjiang Medical University Urumqi China; ^2^ Department of Geriatrics Traditional Chinese Medicine Hospital Affiliated With Xinjiang Medical University Urumqi China

**Keywords:** intracerebral hemorrhage, microglia, NLRP3, pyroptosis, rhein

## Abstract

**Background:**

Intracerebral hemorrhage (ICH) induces severe neuroinflammation and microglial pyroptosis, exacerbating secondary brain injury. Rhein, a natural anthraquinone compound, possesses anti‐inflammatory and neuroprotective properties. However, its effects on microglial pyroptosis and the underlying mechanisms remain unclear.

**Methods:**

A rat microglial (RM) pyroptosis model was established using LPS + ATP induction, followed by rhein intervention and NLRP3 knockdown. Cell proliferation was assessed using CCK‐8, and apoptosis was evaluated through TUNEL staining. ELISA was used to measure the expression levels of inflammatory cytokine. Immunofluorescence staining was performed to label M1/M2 microglia. RT‐qPCR and western blot were used to analyze the expression of NLRP3, ASC, Caspase‐1, GSDMD, PCNA, Cyclin D1, and CDK2. Transmission electron microscopy (TEM) was used to observe pyroptotic bodies. Additionally, a rat ICH model was established, with rhein intervention and NLRP3 knockdown/Caspase‐1 inhibition. Behavioral assessments were conducted using the Y‐maze test and open‐field test. HE staining was performed to examine brain tissue pathology. ELISA was used to measure inflammatory cytokine levels in brain tissue. Immunofluorescence staining analyzed the distribution of M1/M2 microglia. RT‐qPCR and western blot were used to detect pyroptosis‐related proteins, and TEM was used to evaluate pyroptotic body formation.

**Results:**

At the cellular level, rhein significantly promoted microglial proliferation and M2 polarization while inhibiting pyroptosis, inflammatory cytokine expression, M1 polarization, and NLRP3 expression. NLRP3 knockdown further enhanced the protective effects of rhein. At the animal level, ICH model rats exhibited reduced exploratory behavior, exacerbated neuroinflammation, increased pro‐inflammatory cytokine expression, increased M1 microglia, and elevated NLRP3 expression and pyroptosis levels. Rhein intervention significantly alleviated inflammation in ICH rats by reducing the expression of NLRP3 and pyroptosis‐related proteins. NLRP3 knockdown or Caspase‐1 inhibition further enhanced rhein's protective effects.

**Conclusion:**

Rhein alleviates neurological dysfunction following ICH by inhibiting NLRP3 inflammasome activation, reducing microglial pyroptosis, and mitigating neuroinflammation.

## Graphical abstract

Rhein treatment inhibits NLRP3 inflammasome activation, suppresses Caspase‐1/GSDMD‐mediated pyroptosis, reduces pro‐inflammatory cytokine release, promotes M2 polarization, and improves behavioral outcomes of ICH. These findings suggest that rhein protects against ICH‐induced brain injury through the inhibition of NLRP3‐dependent pyroptosis, highlighting its translational potential as a therapeutic candidate for hemorrhagic stroke.

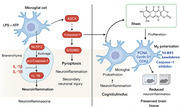

**Abbreviations**
ASC: apoptosis‐associated speck‐like protein containing a CARD
ATP: adenosine triphosphate
CCK‐8: Cell Counting Kit‐8
CDK2: cyclin‐dependent kinase 2
ELISA: enzyme‐linked immunosorbent assay
GSDMD: gasdermin D
HE: hematoxylin and eosin
ICH: intracerebral hemorrhage
IL‐6: interleukin‐6
IL‐18: interleukin‐18
IL‐1β: interleukin‐1 beta
iNOS: inducible nitric oxide synthase
LPS: lipopolysaccharide
NLRP3: NOD‐like receptor family pyrin domain‐containing protein 3
PCNA: proliferating cell nuclear antigen
RM: rat microglia
RT‐qPCR: real‐time quantitative PCR polymerase chain reaction
SD: Sprague‐Dawley
TEM: transmission electron microscopy
TNF‐α: tumor necrosis factor alpha

## 1 Introduction

Stroke is the second leading cause of death and disability worldwide, with intracerebral hemorrhage (ICH) accounting for approximately 10% of all strokes (GBD 2019 Stroke Collaborators 2021). ICH is associated with a high risk of disability and mortality (M. Wang et al. 2024). Survivors of ICH often experience severe neurological deficits, including motor dysfunction and cognitive impairment, imposing a heavy burden on patients, families, and society (Sasongko et al. 2025). Although current treatment strategies have significantly reduced mortality, their efficacy in improving patient prognosis remains limited (Luo et al. 2024). Therefore, exploring the pathological mechanisms of secondary brain injury following ICH and identifying effective pharmacological targets and therapeutic strategies hold significant clinical importance.

Recent studies have shown that secondary inflammatory responses play a key role in the progression of brain injury after ICH. Microglia, as the primary immune cells in the central nervous system, are crucial mediators of neuroinflammation following brain tissue injury (S. Li et al. 2022). The imbalance between M1/M2 microglial polarization is a critical factor contributing to exacerbated neuroinflammation and worsened neurological outcomes after ICH (Bi et al. 2021). Moreover, an emerging form of inflammation‐related programmed cell death, pyroptosis, has attracted considerable attention in the context of cerebrovascular diseases (Jin et al. 2022). The NLRP3 inflammasome (NOD‐like receptor family, pyrin domain‐containing protein 3) is a key regulatory pathway of pyroptosis. Its aberrant activation leads to ASC (apoptosis‐associated speck‐like protein containing a CARD) aggregation, subsequent Caspase‐1 activation, and cleavage of downstream pyroptotic protein gasdermin D (GSDMD), ultimately triggering a robust neuroinflammatory response (He et al. 2025; Song et al. 2022). As a result, targeting the NLRP3 inflammasome and pyroptotic signaling pathways to inhibit neuroinflammation and alleviate brain injury has become a research hotspot in ICH treatment.

Rhein, a natural anthraquinone compound derived from the traditional Chinese medicinal herb *Rheum palmatum* (rhubarb), has been reported to exhibit anti‐inflammatory, anti‐apoptotic, antioxidant, and neuroprotective properties (Y. Wang et al. 2016; X. Li et al. 2019). However, the role of rhein in secondary brain injury following ICH remains unclear, particularly regarding its potential to regulate the NLRP3 inflammasome, inhibit microglial pyroptosis, and improve neurological deficits. Therefore, further investigation into the neuroprotective effects, underlying mechanisms, and therapeutic targets of rhein in ICH has significant clinical translational potential.

Based on this research background, our study aims to elucidate how rhein modulates the NLRP3 inflammasome pathway, inhibits microglial pyroptosis, restores microglial polarization balance, and suppresses neuroinflammatory responses. We aim to provide new theoretical insights and potential pharmacological targets for the clinical treatment of neurological damage following ICH. This study seeks to uncover the molecular mechanisms by which rhein alleviates ICH‐induced brain injury, laying a solid foundation for the development of novel neuroprotective therapeutic strategies.

## 2 Materials and Methods

### 2.1 Cell Culture and Construction of Pyroptosis Model

Rat microglial cells (rat microglia, RM) were purchased from BeNa Culture Collection (China) and cultured in high‐glucose Dulbecco's Modified Eagle Medium (Gibco, USA) supplemented with 10% fetal bovine serum (Gibco) and 1% penicillin‐streptomycin (Gibco). The cells were maintained at 37°C in a humidified incubator with 5% CO_2_. When reaching 80%–90% confluence, the cells were used for experimental treatments.

The microglial pyroptosis model was established by stimulating the cells with lipopolysaccharide (LPS; 10 µg/mL; Sigma‐Aldrich, USA) for 6 h, followed by adenosine triphosphate (ATP; 5 mM; Sigma‐Aldrich) incubation for 30 min. After LPS + ATP stimulation, the cells were treated with rhein (25 µM; Yuanye Bio, China) for 24 h.

For stable NLRP3 gene knockdown, an adenovirus‐mediated shRNA transfection strategy was used. The NLRP3‐targeting shRNA was synthesized by GenePharma (Shanghai, China) and cloned into the recombinant adenoviral expression vector pAV‐U6‐GFP. A scrambled shRNA (sh‐NC) was used as a negative control. The recombinant adenovirus plasmid was transfected into 293T cells (BeNa Culture Collection) using Lipofectamine 2000 (Thermo Fisher, USA). The viral supernatant was collected 48 h post‐transfection. Rat microglial cells were seeded into six‐well plates (2 × 10^5^ cells/well) and infected with adenovirus (multiplicity of infection (MOI) = 100). After 96 h of culture, cells were subjected to further experiments.

The experimental groups were as follows: control, model (LPS + ATP–induced pyroptosis model), control + rhein, model + rhein, model + rhein + sh‐NC, model + rhein + sh‐NLRP3.

### 2.2 Establishment and Intervention of Acute ICH Model in Rats

Healthy male Sprague‐Dawley (SD) rats weighing 200–250 g were obtained from Xinjiang Medical University Laboratory Animal Center. All experiments were conducted in accordance with ethical guidelines for animal research and were approved by the Animal Ethics Committee of Xinjiang Medical University (No. IACUC‐20210301‐25). Before the experiments, rats were acclimated for 7 days in a standard laboratory environment.

Rats were anesthetized with isoflurane (induction: 5%, maintenance: 2%) and maintained under anesthesia using a ventilator. Using a stereotactic frame, an injection was made at coordinates 1.4 mm posterior to the bregma and 3.2 mm lateral to the right. A microinjection pump was used to slowly inject Type VII collagenase (5 U, 0.2 U/µL; Sangon Biotech, China) to induce ICH. Sham‐operated rats underwent the same procedures but received an injection of normal saline instead of collagenase.

The 50 SD rats were randomly divided into the following groups: sham group, ICH group, sham + rhein group, ICH + rhein group, sham + rhein + sh‐NLRP3 group, ICH + rhein + sh‐NLRP3 group, sham + rhein + Ad‐NC group, ICH + rhein + Ad‐NC group, ICH + caspase‐1 inhibitor group, and ICH + rhein + caspase‐1 inhibitor group. For drug intervention, rats received rhein (0.865, 1.73, and 3.46 mg/kg, i.p.) once daily for 7 days. Additionally, 1 h after ICH induction, rats received Caspase‐1 inhibitor (VX‐765, 25 mg/kg, via tail vein injection, once daily for 7 days). Rats were received sh‐NLRP3 adenovirus (5 × 10^9^ pfu per rat, via tail vein injection, once daily for 7 days). On postoperative day 7, rats were anesthetized with 1% sodium pentobarbital (50 mg/kg, i.p.), and blood and brain tissue samples were collected after euthanasia.

### 2.3 Behavioral Tests

Post‐surgery, rats underwent the Y‐maze test and open‐field test. The Y‐maze was placed in a quiet, evenly lit laboratory. One day before the experiment, rats were allowed to explore the maze freely for 5 min to adapt to the environment. During the test, each rat was tested in a fixed order. The rat was placed in the central area, always facing Arm A, and allowed to explore freely for 5 min. The movement trajectory was recorded in real time, and path tracking and data analysis were performed.

For the open‐field test, rats were allowed to freely explore the open‐field box for 5 min 1 day before the experiment for adaptation. During the test, each rat was tested in a fixed sequence. The rat was placed in the central area of the open‐field box and allowed to explore freely for 5 min. The movement trajectory was recorded in real time, and path tracking and data analysis were conducted.

### 2.4 Cell Proliferation Detection

The Cell Counting Kit‐8 (CCK‐8; Beyotime, China) was used to assess cell proliferation ability. RM cells were seeded into 96‐well plates (5 × 10^4^ cells per well) and incubated with different treatments. After incubation at 37°C for 24, 36, 48, and 72 h, 10 µL of CCK‐8 solution was added to each well, followed by an additional 2‐h incubation. Absorbance (optical density) was measured at 450 nm using a microplate reader.

### 2.5 Cell Pyroptosis and Apoptosis Detection

Pyroptotic body formation was examined using transmission electron microscopy (TEM). Cell and brain tissue samples were fixed with 2.5% glutaraldehyde, dehydrated using graded ethanol, embedded in epoxy resin, and ultra‐thin sectioned (70 nm). The sections were stained with uranyl acetate and lead citrate and observed under a TEM to assess pyroptosis‐related ultrastructural changes.

Apoptosis of microglial cells was assessed using a TUNEL apoptosis detection kit. TUNEL staining was performed according to the manufacturer's instructions. Fluorescence signals were observed under a fluorescence microscope (Leica, Germany), and the percentage of TUNEL‐positive cells was calculated.

Apoptosis was further analyzed using Annexin V‐FITC/PI double staining. A brain tissue cell suspension was prepared and adjusted to 1 × 10^6^ cells/mL. Note that 100 µL of cell suspension and 100 µL of RM cells were mixed separately with 100 µL of Annexin V binding buffer. Then, 5 µL of Annexin V‐FITC and 5 µL of PI were added and incubated in the dark for 15 min. The cells were then centrifuged at 1000 rpm for 5 min, the supernatant was discarded, and the cells were resuspended in phosphate buffered saline (PBS) before being analyzed using flow cytometry. A total of 10,000 events were recorded, and data were analyzed using the FlowJo software.

### 2.6 Enzyme‐Linked Immunosorbent Assay

The levels of interleukin‐1 beta (IL‐1β), interleukin‐18 (IL‐18), tumor necrosis factor alpha (TNF‐α), interleukin‐6 (IL‐6), and inducible nitric oxide synthase (iNOS) in cell culture supernatants and rat serum samples were measured using enzyme‐linked immunosorbent assay (ELISA) kits (Biosharp, China). The assays were performed strictly according to the manufacturer's instructions. Absorbance was measured at 450 nm using a microplate reader, and cytokine concentrations were calculated accordingly.

### 2.7 Immunofluorescence Staining for Microglial Polarization

Immunofluorescence double staining was performed to assess M1/M2 microglial polarization at both the tissue and cellular levels. Brain tissue samples and cells were fixed with 4% paraformaldehyde. Tissue samples were dehydrated, cleared, and embedded in paraffin. The paraffin blocks were sectioned into 5‐µm thick continuous slices. After permeabilization with 0.3% Triton X‐100, cells and tissue sections were incubated with 5% bovine serum albumin (BSA) at room temperature for 1 h. Samples were then incubated overnight at 4°C with primary antibodies: Iba1 and CD86 (for M1 microglia) or Iba1 and CD206 (for M2 microglia) (1:200; Abcam, UK). After washing with PBS, samples were incubated with secondary antibodies (1:500; Abcam). Cell nuclei were counterstained with 4',6‐Diamidino‐2‐Phenylindole (DAPI), and images were captured using a fluorescence microscope.

### 2.8 Real‐Time Quantitative PCR Polymerase Chain Reaction

The total RNA was extracted from cells and brain tissues using TRIzol reagent (Invitrogen, USA). Reverse transcription was performed using the PrimeScript RT Reagent Kit (Takara, Japan) to synthesize cDNA. qPCR analysis was conducted using SYBR Green qPCR Mix (Takara). β‐Actin was used as an internal reference, and relative gene expression levels were calculated using the 2^−ΔΔCt^ method. Primer sequences are listed in Table S1.

### 2.9 Western Blot

Total protein was extracted from cells and brain tissues using RIPA lysis buffer containing 1% protease inhibitor. Protein concentration was determined using a BCA protein assay kit (Beyotime). Equal amounts of protein (20 µg per sample) were separated by SDS‐PAGE and subsequently transferred onto PVDF membranes. The membranes were blocked with 5% skim milk for 1 h. The membranes were incubated overnight at 4°C with primary antibodies. After washing, the membranes were incubated with horseradish peroxidase (HRP)‐conjugated secondary antibodies for 2 h. Protein bands were visualized using ECL chemiluminescence detection, with β‐actin used as an internal control. Protein band intensity was quantified using the ImageJ software.

### 2.10 Hematoxylin‐Eosin Staining and Immunohistochemistry

Brain tissue samples were fixed in 4% paraformaldehyde for 24 h. After dehydration and clearing, the tissues were embedded in paraffin. The paraffin blocks were sectioned into 5‐µm thick slices and deparaffinized with xylene, followed by dehydration using 100%, 95%, 90%, 80%, and 70% ethanol solutions. The sections were stained with hematoxylin for 3 min, then immersed in eosin solution for 2 min. Slides were mounted with neutral resin and observed under a light microscope.

For immunohistochemistry analysis, tissue sections were subjected to antigen retrieval in sodium citrate buffer (pH 6.0) for 15 min. After blocking with 5% goat serum at room temperature for 30 min, sections were incubated overnight at 4°C with rabbit anti‐GSDMD primary antibody (1:200; Abcam). The next day, sections were incubated with HRP‐conjugated goat anti‐rabbit IgG secondary antibody (1:500; Abcam) at room temperature for 1 h. Color development was performed using DAB substrate solution for 3 min, and the staining was observed under a light microscope. Cell nuclei were counterstained with hematoxylin for 1 min. The expression of GSDMD in tissue samples was evaluated microscopically.

### 2.11 Statistical Analyses

All statistical analyses were conducted using GraphPad Prism software 10.0. Data are presented as mean ± standard deviation (mean ± SD). Student's *t*‐test was used for comparisons between the two groups, while one‐way analysis of variance with LSD post hoc test was applied for multiple group comparisons. Each assay was independently repeated at least three times. For in vivo experiments, five rats were included per group. Behavioral tests, histological assessments, and western blot quantifications were performed by investigators blinded to group allocation. *p* < 0.05 was considered statistically significant.

## 3 Results

### 3.1 Effects of Rhein on LPS + ATP–Induced Microglial Proliferation and Pyroptosis

Transfection with shRNA‐NLRP3 significantly reduced NLRP3 expression in microglial cells (Figure S1A,B). In the LPS + ATP–treated group (Model), the mRNA level of NLRP3 was significantly increased, while shRNA‐NLRP3 transfection effectively reduced its expression (Figure S1C).

**FIGURE 1:**
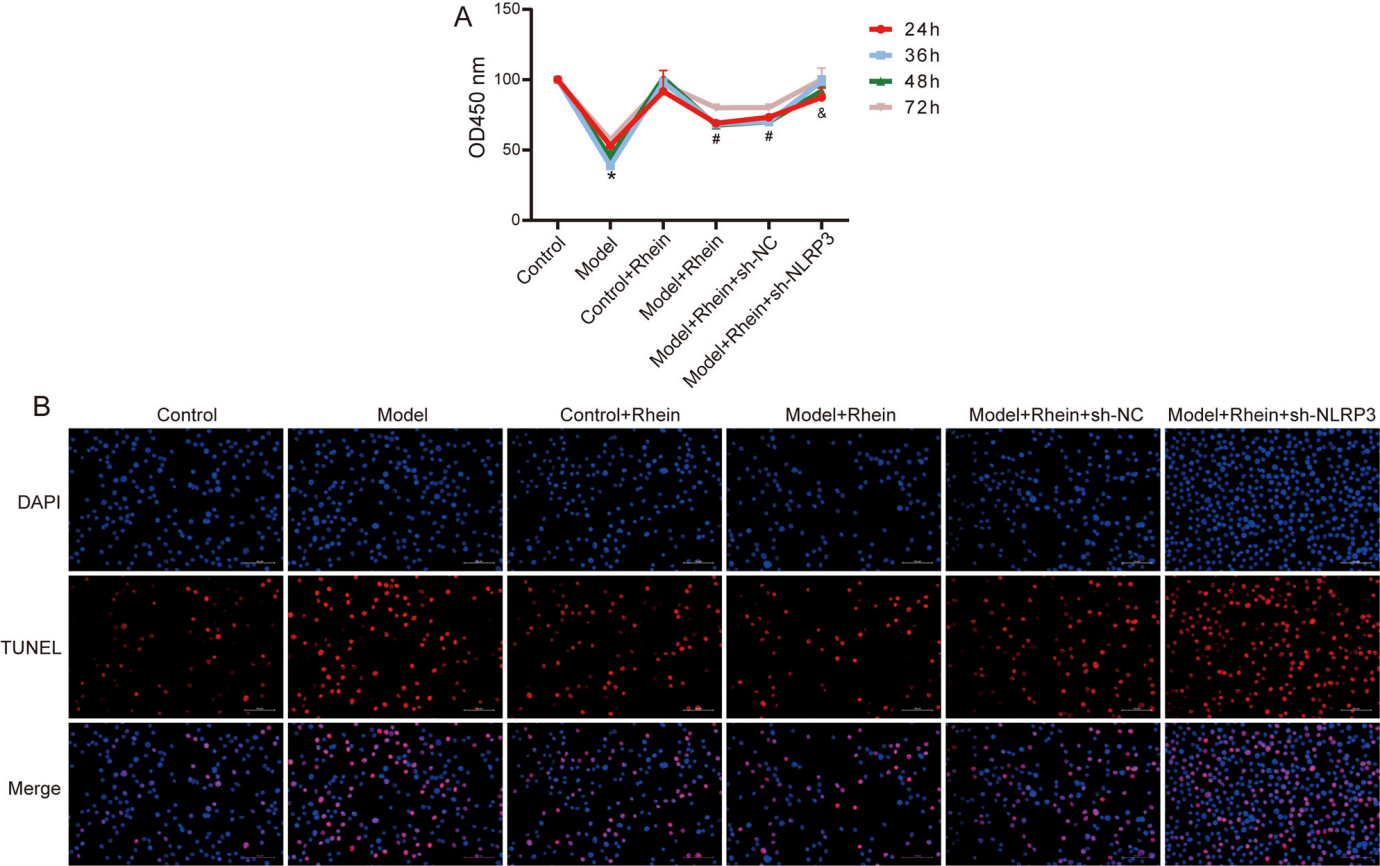
The effect of rhein on proliferation and apoptosis of microglia. (A) CCK‐8 detects cell viability in different groups. (B) Cell apoptosis in different groups was detected using TUNEL staining. *p < 0.05 compared to control; #*p* < 0.05 compared to model; &*p* < 0.05 compared to model + rhein + sh‐NC.

The CCK‐8 assay results (Figure 1A) showed that compared to the control group, microglial cell viability was significantly reduced in the LPS + ATP–treated group (*p* < 0.05). However, following rhein intervention, cell proliferation was significantly enhanced (*p* < 0.05). Moreover, after shRNA‐NLRP3 transfection, rhein‐treated cells exhibited further promotion of proliferation (*p* < 0.05).

TUNEL staining results (Figure 1B) further confirmed that LPS + ATP induction significantly increased the proportion of TUNEL‐positive cells (*p* < 0.05) compared to the control group. After rhein treatment, the percentage of TUNEL‐positive cells significantly decreased (*p* < 0.05). Furthermore, in the NLRP3 knockdown group, rhein further reduced the proportion of TUNEL‐positive cells (*p* < 0.05), indicating that the NLRP3 signaling pathway is involved in the anti‐apoptotic effects of rhein.

**FIGURE 2:**
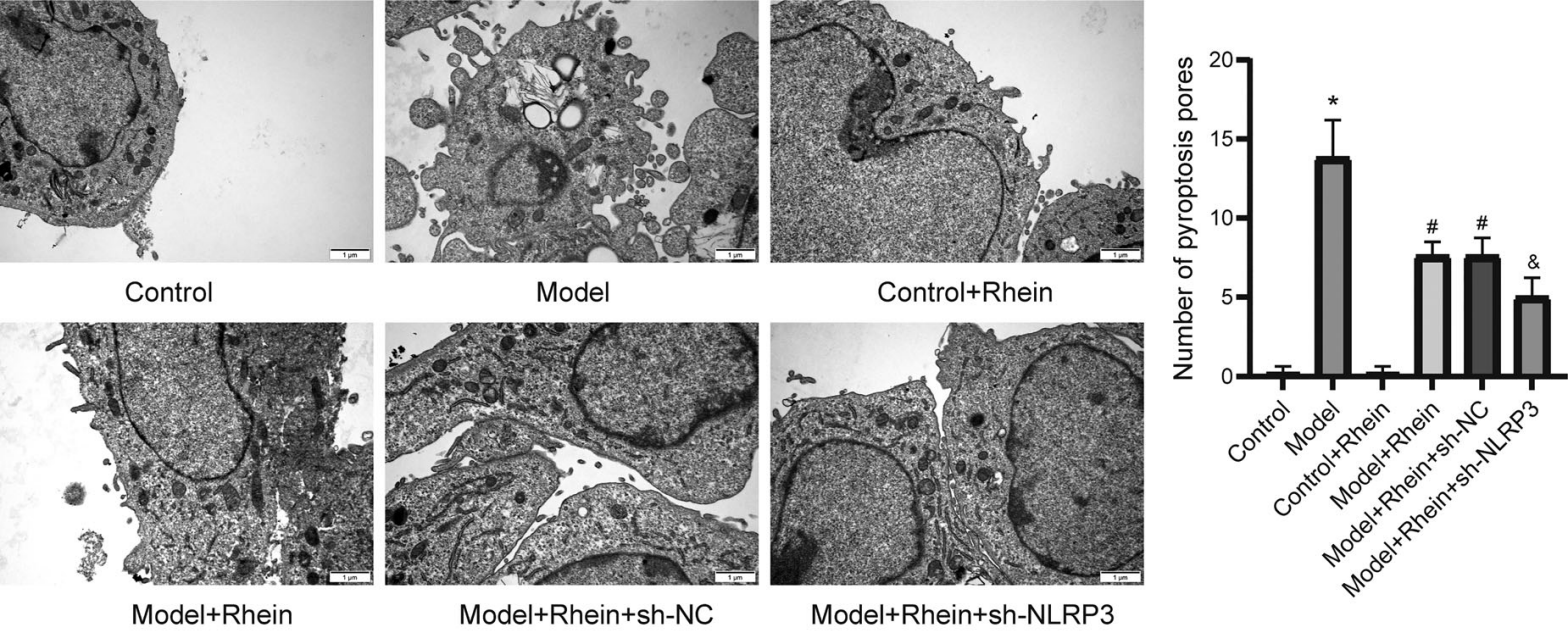
Observation of pyroptosis using transmission electron microscopy. Bar = 1 µm. *p < 0.05 compared to control; #*p* < 0.05 compared to model; &*p* < 0.05 compared to model + rhein + sh‐NC.

TEM observations (Figure 2) showed that control microglial cells maintained an intact morphology with no apparent pyroptotic features. In contrast, LPS + ATP–induced microglia displayed significant plasma membrane bubbling, cell swelling, and abundant formation of pyroptotic bodies. After rhein treatment, the number of pyroptotic bodies was markedly reduced, and the membrane structure remained more intact. Additionally, in the NLRP3 knockdown group, rhein's protective effect was further enhanced, leading to greater improvements in pyroptotic morphology and a significant reduction in pyroptotic bodies.

### 3.2 Rhein Regulates Microglial Polarization and the Secretion of Inflammatory Cytokines

**FIGURE 3:**
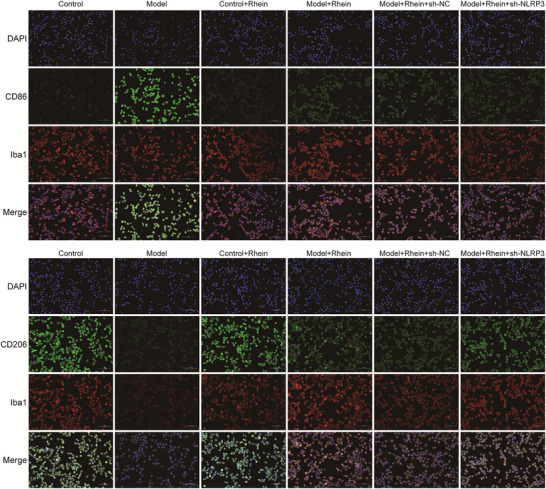
Rhein promotes M2 polarization and inhibits M1 polarization. Bar = 100 µm.

Immunofluorescence staining (Figure 3) demonstrated that in the control group, the number of M1 microglial cells (CD86‐positive cells) was relatively low. However, LPS + ATP stimulation significantly increased M1 microglial cells, while the number of M2 microglial cells (CD206‐positive cells) markedly decreased. Rhein intervention resulted in a significant decrease in M1 microglial cells and an increase in M2 microglial cells. Moreover, NLRP3 knockdown further enhanced the effects of rhein by promoting M2 polarization and suppressing M1 polarization.

**FIGURE 4:**
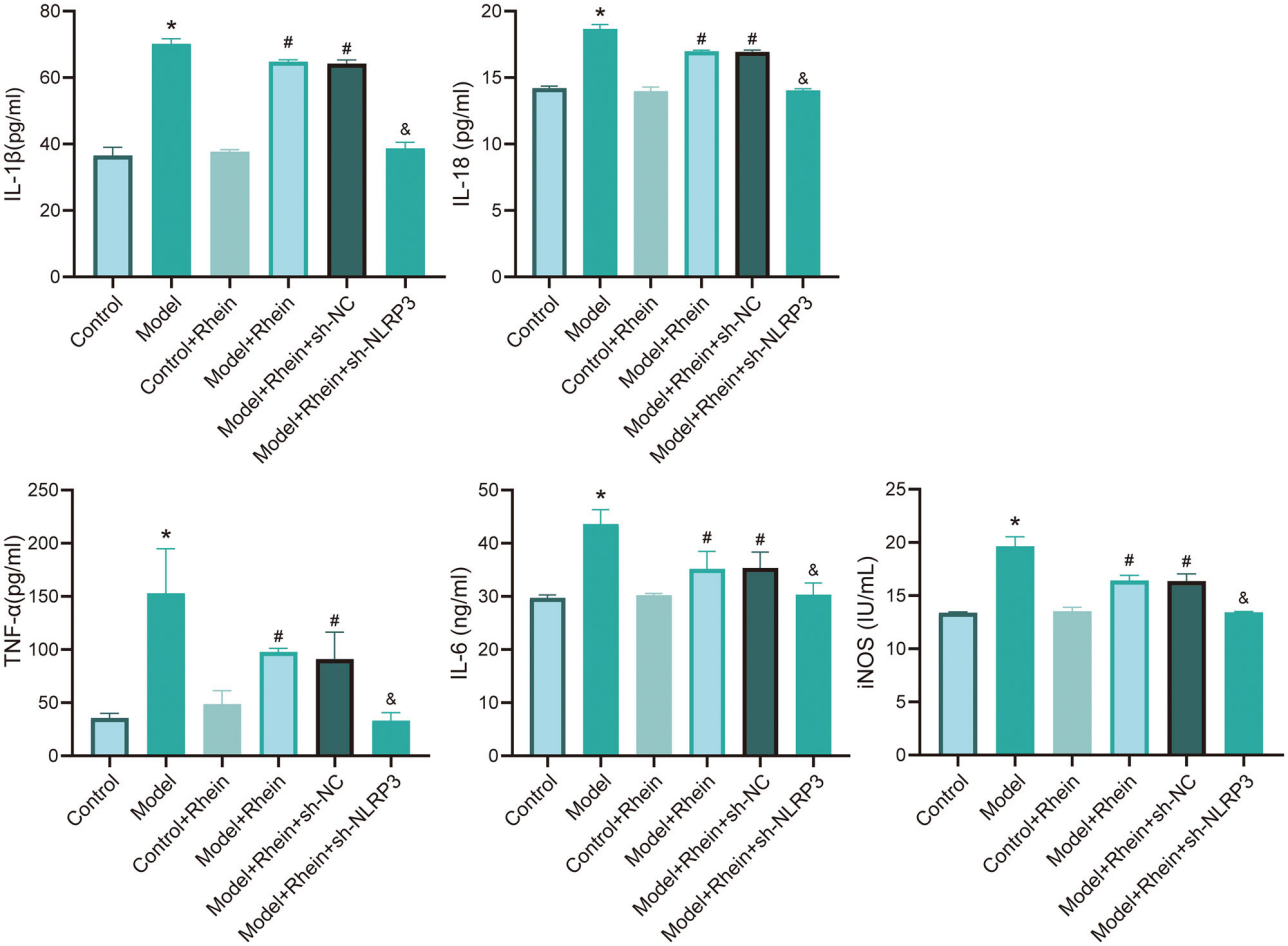
ELISA detection of inflammatory factors. *p < 0.05 compared to control; #*p* < 0.05 compared to model; &*p* < 0.05 compared to model + rhein + sh‐NC.

ELISA analysis was conducted to detect the levels of inflammatory cytokines IL‐1β, IL‐18, TNF‐α, IL‐6, and iNOS in the cell culture supernatant. The results showed that compared to the control group, LPS + ATP treatment significantly increased the levels of these inflammatory cytokines. However, rhein intervention significantly reduced their expression. Furthermore, NLRP3 knockdown further enhanced the anti‐inflammatory effects of rhein, leading to a further reduction in inflammatory cytokine levels (Figure 4).

### 3.3 Rhein Reduces the Expression of NLRP3 and Pyroptosis‐Related Proteins

**FIGURE 5:**
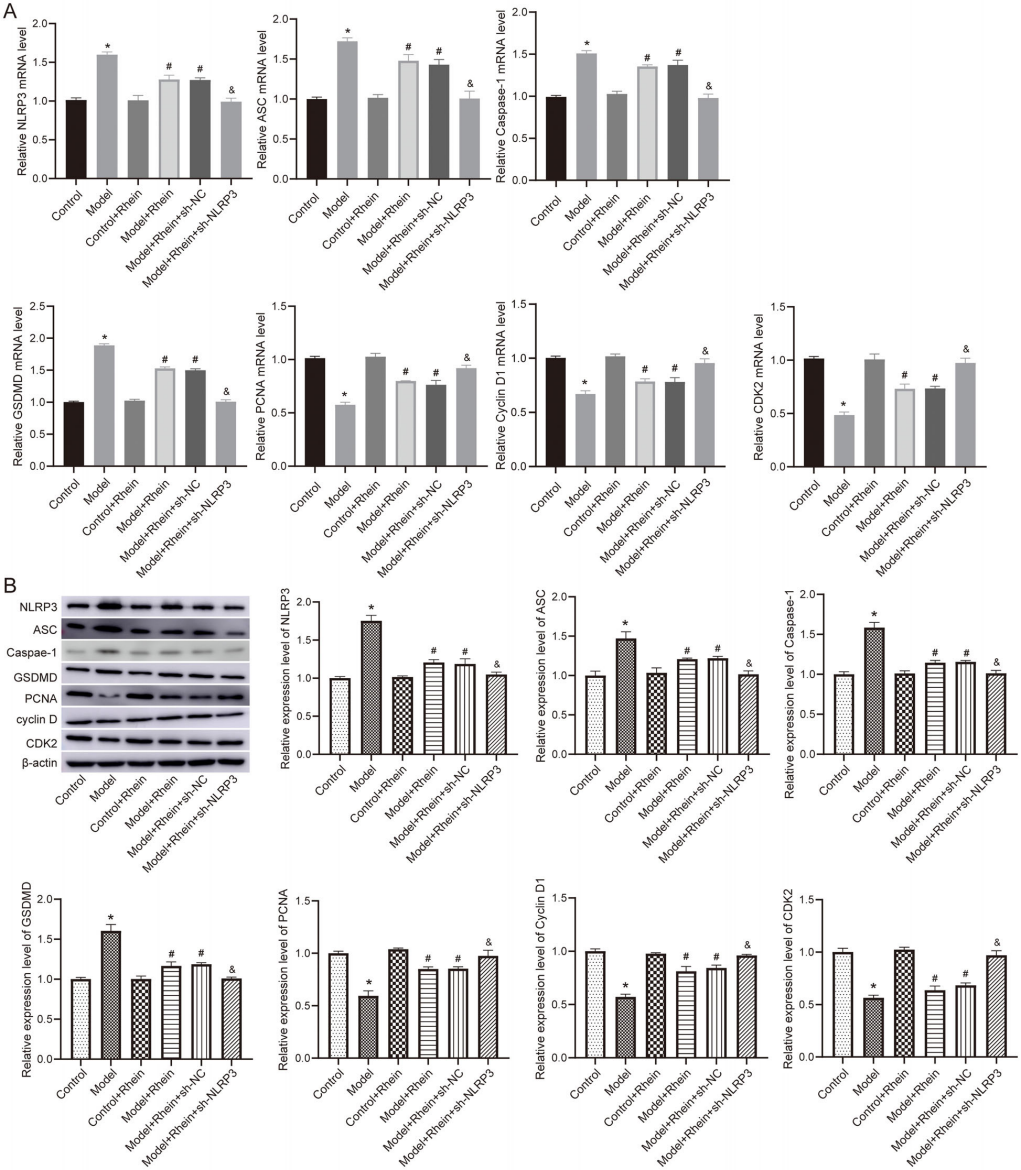
Expression of pyroptosis‐related proteins detected by RT‐qPCR and western blot. (A) The mRNA levels detected by RT‐qPCR. (B) The protein levels detected by western blot. *p < 0.05 compared to control; #*p* < 0.05 compared to model; &*p* < 0.05 compared to model + rhein + sh‐NC.

Real‐time quantitative PCR polymerase chain reaction (RT‐qPCR) (Figure 5A) and western blot (Figure 5B) analyses showed that compared to the control group, LPS + ATP stimulation significantly increased the mRNA and protein levels of NLRP3, ASC, Caspase‐1, and GSDMD. However, rhein treatment markedly reduced the expression levels of these proteins. Furthermore, NLRP3 knockdown led to a further significant downregulation of pyroptosis‐related protein expression, suggesting that NLRP3 plays a key role in the anti‐pyroptotic effects of rhein.

Additionally, after LPS + ATP treatment, the mRNA and protein levels of proliferating cell nuclear antigen (PCNA), Cyclin D1, and cyclin‐dependent kinase 2 (CDK2) were significantly decreased. However, rhein intervention significantly upregulated the expression of these genes and proteins, and NLRP3 knockdown further enhanced the proliferative effects of rhein.

### 3.4 Rhein Improves Neurological Function in ICH Rats

Y‐maze and open‐field tests were conducted at 24, 48, and 72 h post‐modeling to evaluate the success of the ICH model. The results confirmed that the ICH model was successfully established 48 h after type VII collagenase injection (Figure S2). Rats with ICH were treated with 0.865, 1.73, and 3.46 mg/kg doses of rhein via intraperitoneal injection once daily. Y‐maze and open‐field tests indicated that 1.73 mg/kg was the optimal therapeutic dose of rhein (Figure S3).

**FIGURE 6:**
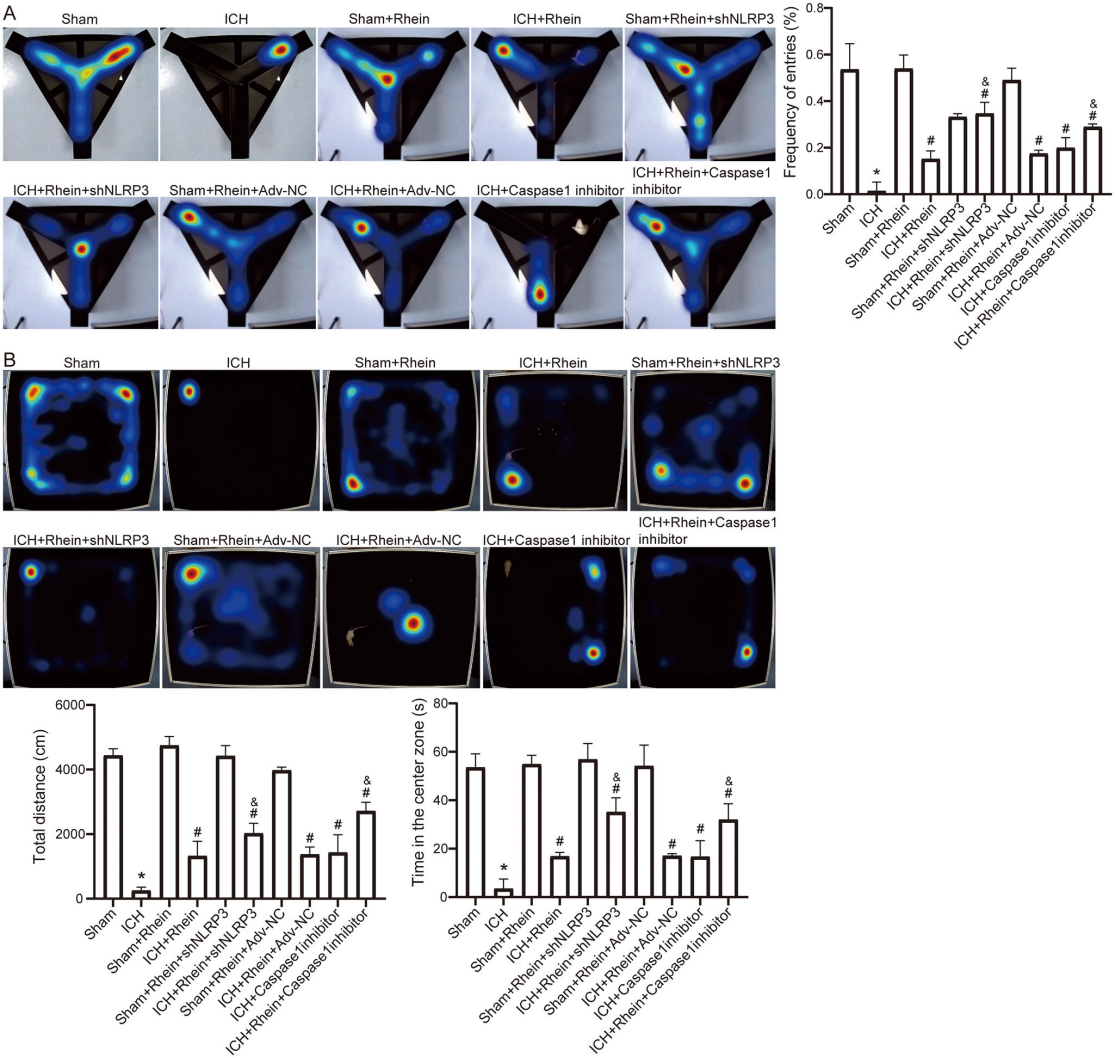
The improvement effect of rhein on the behavior of ICH rat model. (A) Y‐maze experiment results. (B) Open‐field experiment results. *p < 0.05 compared to sham; #*p* < 0.05 compared to ICH; & *p* < 0.05 compared to ICH + Rhein.

On postoperative day 7, the Y‐maze test results (Figure 6A) showed that the alternation rate in the ICH group was significantly reduced, indicating severe cognitive impairment caused by ICH. After rhein treatment, the alternation rate significantly improved. Moreover, in the NLRP3 knockdown or Caspase‐1 inhibitor intervention groups, the alternation rate was further increased, suggesting an enhanced recovery of neurological function.

The open‐field test results (Figure 6B) showed that in the ICH group, total movement distance and time spent in the central area were significantly reduced (*p* < 0.05), indicating obvious motor dysfunction and anxiety‐like behavior. After rhein treatment, the total movement distance and central area exploration time were significantly increased. Furthermore, in the NLRP3 knockdown or Caspase‐1 inhibition groups, the neuroprotective effects of rhein were further enhanced.

### 3.5 Rhein Reduces Inflammation Levels in the Brain Tissue of ICH Rats

**FIGURE 7:**
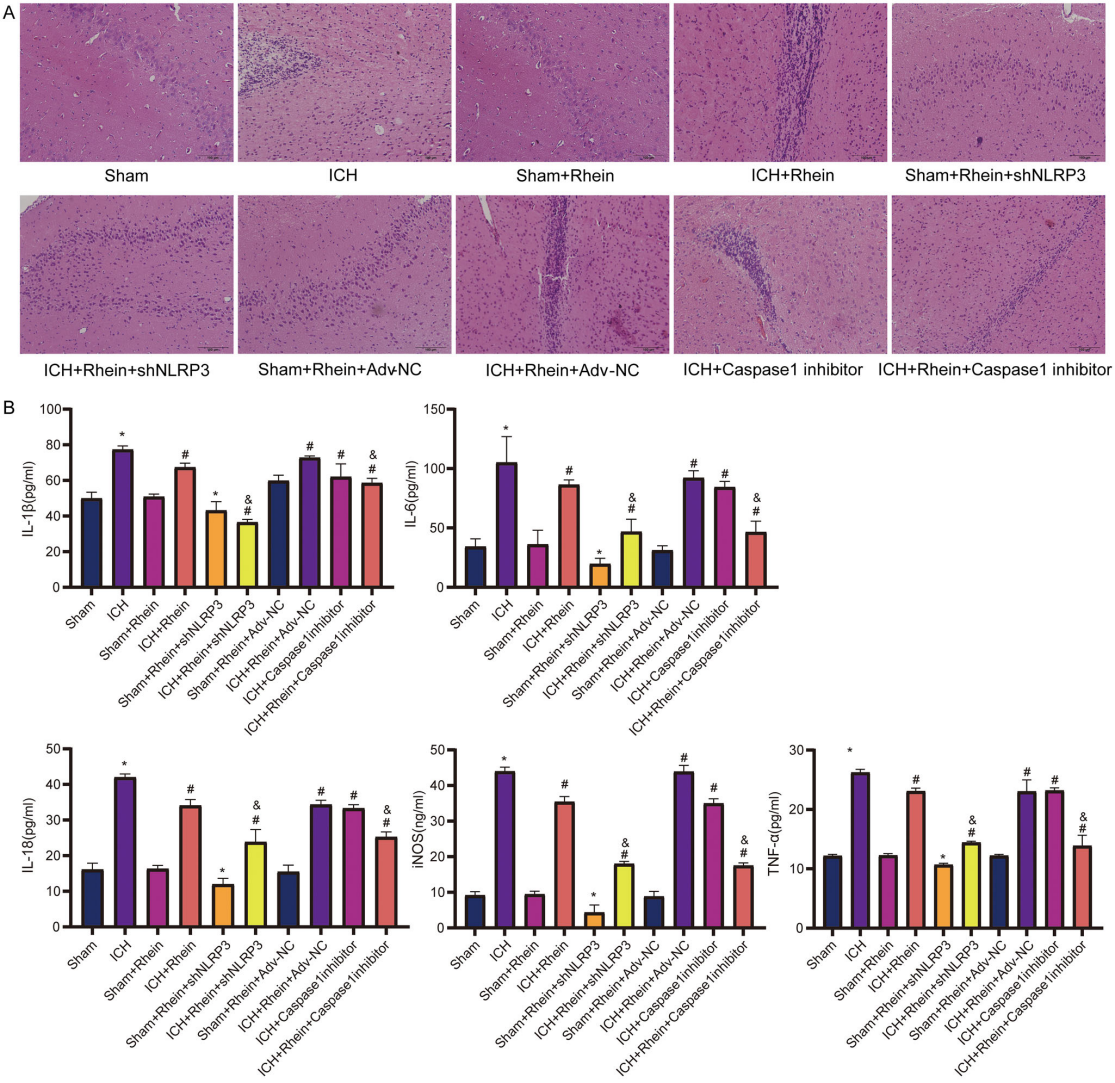
Inflammation detection of rat brain tissue. (A) HE staining observation of brain tissue pathology. (B) Detection of inflammatory cytokine levels in brain tissue. *p < 0.05 compared to sham; #*p* < 0.05 compared to ICH; &*p* < 0.05 compared to ICH + rhein.

Hematoxylin‐eosin (HE) staining results (Figure 7A) showed that in the ICH group, brain tissues exhibited significant inflammatory cell infiltration and tissue edema. However, rhein intervention markedly improved the pathological damage, including reduced tissue edema and decreased inflammatory cell infiltration. Furthermore, NLRP3 knockdown and Caspase‐1 inhibition further alleviated pathological damage in the brain tissue after ICH.

ELISA results (Figure 7B) demonstrated that compared to the sham group, the levels of IL‐1β, IL‐18, TNF‐α, IL‐6, and iNOS were significantly increased in the ICH group. After rhein treatment, the expression levels of these inflammatory cytokines were significantly reduced. Additionally, NLRP3 knockdown and Caspase‐1 inhibition further enhanced rhein's anti‐inflammatory effects, leading to a more pronounced reduction in inflammatory cytokine levels.

The immunofluorescence staining results (Figures S4 and S5) revealed that compared to the sham group, the ICH group showed a significant increase in M1 microglial cells and a reduction in M2 microglial cells. However, after rhein intervention, M1 microglial cells decreased, while M2 microglial cells increased. NLRP3 knockdown and Caspase‐1 inhibition further amplified this effect, suggesting that rhein promotes M2 microglial polarization while inhibiting M1 microglial polarization.

### 3.6 Rhein Reduces Pyroptosis Levels in the Brain Tissue of ICH Rats

**FIGURE 8:**
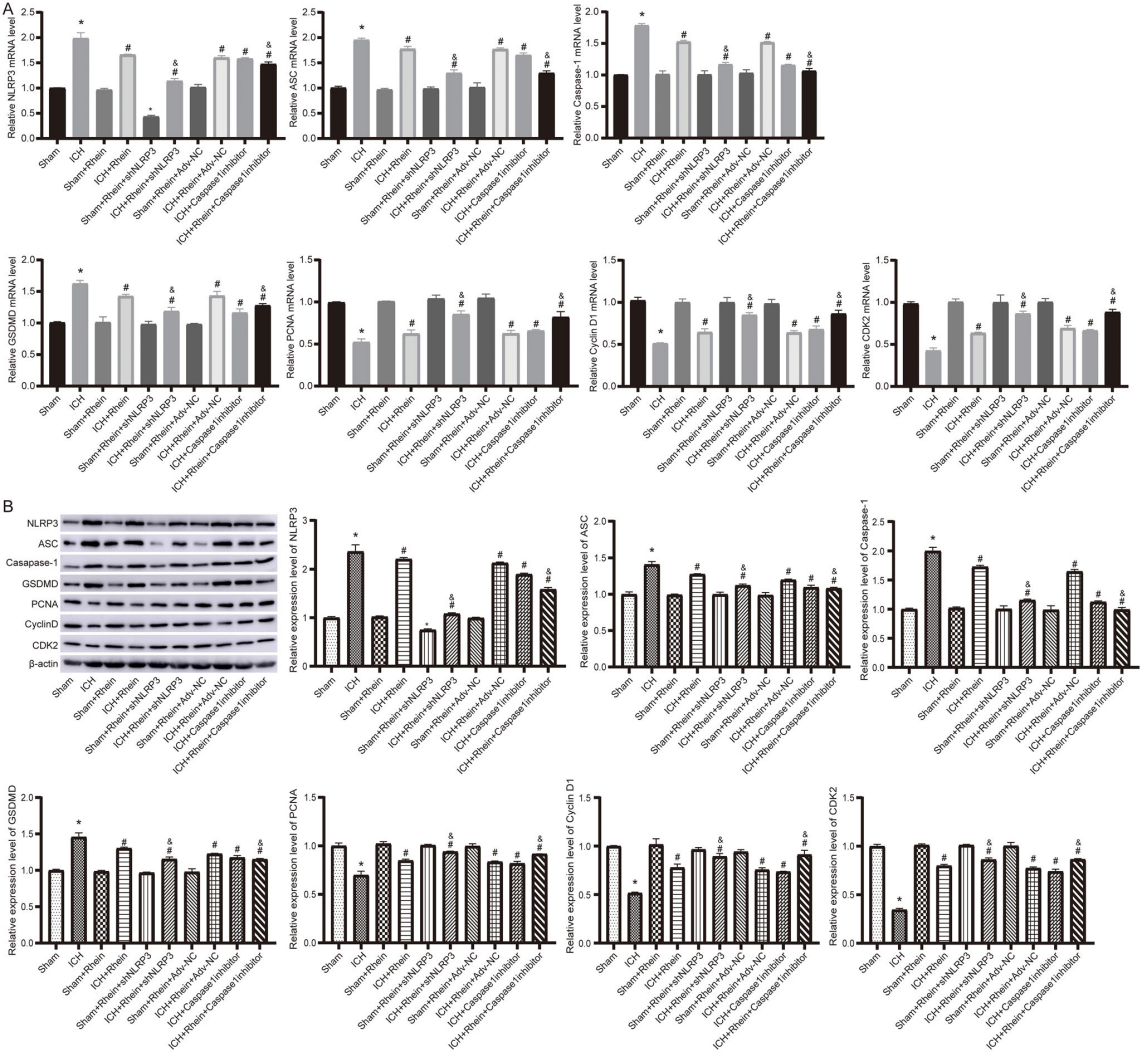
Detection of pyroptosis‐related proteins in brain tissue. (A) The mRNA levels detected by RT‐qPCR. (B) The protein levels detected by western blot. *p < 0.05 compared to sham; #*p* < 0.05 compared to ICH; &*P* < 0.05 compared to ICH + rhein.

RT‐qPCR (Figure 8A) and western blot (Figure 8B) were used to detect the expression of pyroptosis‐related proteins (NLRP3, ASC, Caspase‐1, GSDMD) and proliferation‐related proteins (PCNA, Cyclin D1, CDK2). The results showed that compared to the sham group, the ICH group exhibited significantly increased expression of pyroptosis‐related proteins and decreased expression of proliferation‐related proteins. After rhein intervention, the expression levels of pyroptosis‐related proteins were significantly downregulated, while proliferation‐related proteins were significantly upregulated. Moreover, NLRP3 knockdown and Caspase‐1 inhibition further enhanced the effects of rhein.

**FIGURE 9:**
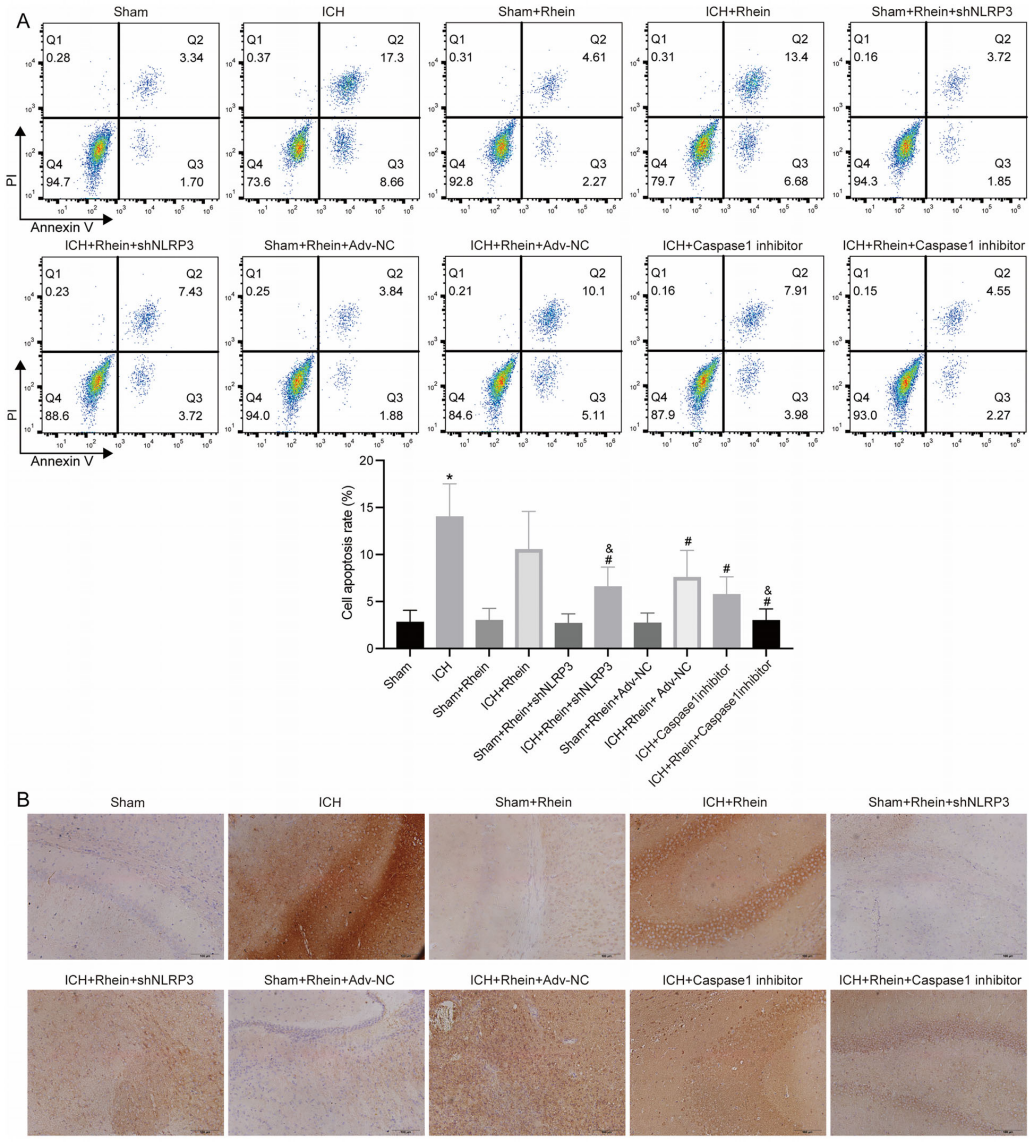
Detection of cell apoptosis and GSDMD expression. (A) The levels of cell apoptosis in brain tissue were detected by flow cytometry. (B) GSDMD expression levels in brain tissue were detected by immunohistochemistry. *p < 0.05 compared to sham; #*p* < 0.05 compared to ICH; &*p* < 0.05 compared to ICH + rhein.

The apoptosis detection results (Figure 9A) indicated that compared to the sham group, apoptosis levels were significantly increased in the ICH group. After rhein treatment, apoptosis levels were significantly reduced, and NLRP3 knockdown or Caspase‐1 inhibition further enhanced the anti‐apoptotic effects of rhein.

The immunohistochemistry results (Figure 9B) showed that compared to the sham group, the expression of GSDMD was significantly increased in the ICH group. However, after rhein treatment, GSDMD expression was reduced, and NLRP3 knockdown or Caspase‐1 inhibition further suppressed GSDMD expression.

**FIGURE 10:**
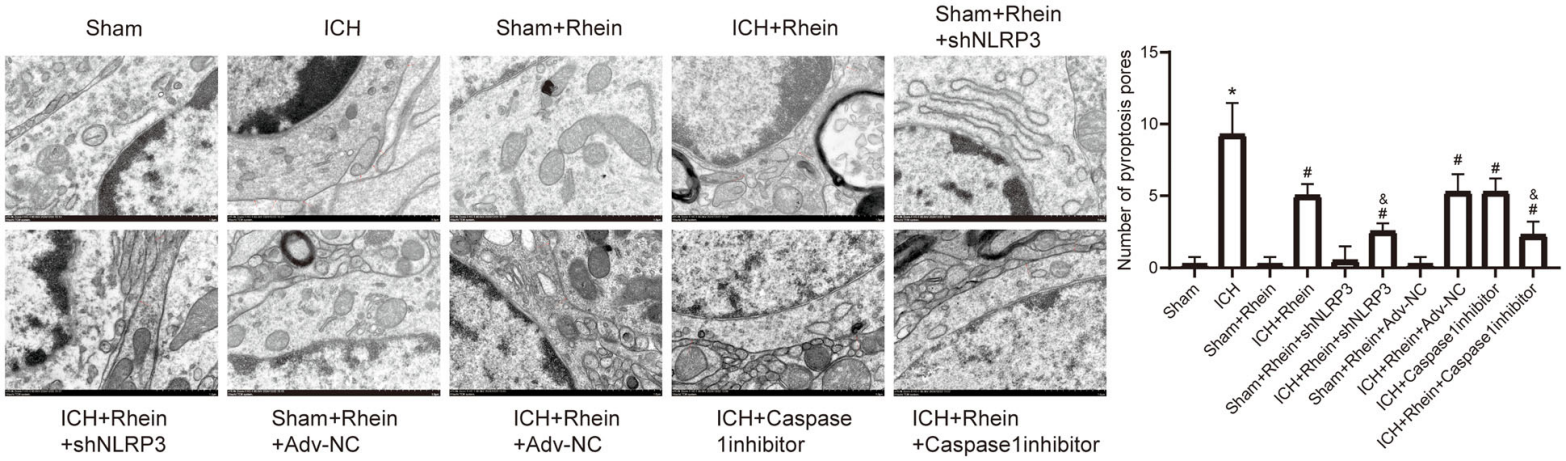
Observation of pyroptosis in brain tissue using transmission electron microscopy. Bar = 1 µm. *p < 0.05 compared to sham; #*p* < 0.05 compared to ICH; &*p* < 0.05 compared to ICH + rhein.

TEM observations (Figure 10) revealed that compared to the sham group, the number of pyroptotic bodies was significantly increased in the brain tissues of the ICH group. After rhein intervention, the formation of pyroptotic bodies was significantly reduced, and NLRP3 knockdown or Caspase‐1 inhibition further enhanced this effect.

## 4 Discussion

ICH is a severe cerebrovascular disease with high disability and mortality rates, posing a serious threat to patients' lives and placing a heavy burden on families and society (J. Yao et al. 2025). Current research suggests that secondary brain injury following ICH is closely associated with massive neuronal loss, neuroinflammatory responses, abnormal microglial activation, and secondary pyroptosis (Lei et al. 2023). Therefore, exploring its pathological mechanisms and identifying effective therapeutic strategies hold great clinical significance. In this study, we innovatively discovered that rhein significantly alleviates microglial pyroptosis and neuroinflammatory responses by inhibiting NLRP3 inflammasome activation, thereby improving neurological dysfunction in ICH rats. The collagenase‐induced ICH rat model is widely accepted and reproducible, as it closely mimics the disruption of the vascular basal lamina and the subsequent inflammatory cascade observed in clinical ICH (Heidarzadegan et al. 2022). Additionally, as a naturally derived small molecule, rhein exhibits low toxicity and favorable pharmacokinetic properties (G. M. Li et al. 2021). Although previous studies have reported that rhein can inhibit NLRP3‐mediated pyroptosis in ischemic models and other inflammatory diseases (Long et al. 2023; Zhou et al. 2022), our study provides novel evidence in the context of ICH. We demonstrated that rhein alleviates ICH‐induced neurological dysfunction by inhibiting NLRP3 inflammasome activation and microglial pyroptosis, and these effects were further validated in vivo through NLRP3 knockdown and Caspase‐1 inhibition.

Pyroptosis is an inflammation‐associated form of programmed cell death that has been demonstrated to play a critical role in the pathological process of ICH (Song et al. 2022). Studies have shown that excessive activation and pyroptosis of microglial cells lead to the release of large amounts of pro‐inflammatory cytokines, exacerbating neuroinflammation and worsening brain injury (R. Ding, Liu, et al. 2022). In this study, we established a classic microglial pyroptosis model using LPS + ATP and found that LPS + ATP treatment significantly inhibited cell proliferation and induced prominent pyroptotic features, including pyroptotic body formation and high expression of the NLRP3 signaling pathway, consistent with previous literature (Tang et al. 2019). Rhein, a natural anthraquinone compound, has been reported to exhibit potent anti‐inflammatory and neuroprotective properties (Liu et al. 2023). Our study further confirmed that rhein significantly improves cell viability, reduces apoptosis, and decreases pyroptotic body formation as observed via TEM, suggesting that rhein may protect microglial cells by inhibiting pyroptosis.

This study also found that NLRP3 gene knockdown further enhanced the inhibitory effect of rhein on microglial pyroptosis. Previous studies have demonstrated that the NLRP3 inflammasome plays a crucial role in the pyroptosis process, and targeting NLRP3 to regulate microglial pyroptosis can alleviate neuroinflammation‐related damage (Wu et al. 2022). Therefore, it is speculated that rhein may inhibit the NLRP3 signaling pathway to block the activation of Caspase‐1 and GSDMD, thereby suppressing microglial pyroptosis. These findings suggest that rhein exerts its protective effects by modulating the NLRP3 signaling pathway to inhibit pyroptosis and maintain cellular viability.

Microglial activation is a key event in the progression of ICH. Activated microglia exhibit two polarization states: the pro‐inflammatory M1 phenotype and the anti‐inflammatory M2 phenotype (Lan et al. 2017). M2 microglial polarization is particularly crucial for controlling neuroinflammation and promoting neuroprotection (Gong et al. 2025). Immunofluorescence results in this study demonstrated that ICH significantly induced M1 polarization while reducing M2 microglial cell numbers. However, rhein effectively reversed this imbalance by increasing M2 polarization and inhibiting M1 polarization. Moreover, NLRP3 knockdown further enhanced M2 polarization, suggesting that the NLRP3 pathway may be an important regulatory signal for microglial polarization (Chen et al. 2020). Based on these findings, it is hypothesized that rhein promotes the shift of microglial polarization toward the anti‐inflammatory M2 phenotype by inhibiting the NLRP3 signaling pathway, thereby reducing the severity of neuroinflammation following ICH.

Additionally, this study found that rhein significantly reduced the levels of pro‐inflammatory cytokines IL‐1β, IL‐18, TNF‐α, IL‐6, and iNOS in both microglial culture supernatants and brain tissues. Moreover, NLRP3 knockdown further enhanced the anti‐inflammatory effects of rhein. These findings further indicate that rhein negatively regulates the NLRP3 signaling pathway, effectively inhibiting microglia‐mediated neuroinflammatory responses and exerting neuroprotective effects (Gu et al. 2022; S.‐T. Yao et al. 2017).

Neurological dysfunction following ICH, including motor impairment, cognitive decline, and emotional abnormalities, is a major factor contributing to poor prognosis in ICH patients (Puy et al. 2022). Neurofunctional deficits after ICH are closely linked to inflammation and pyroptosis (Z. Ding, Zhong, et al. 2022). In this study, rhein significantly improved cognitive function and anxiety‐like behavior in ICH model rats while reducing brain tissue inflammation and pyroptosis‐related protein expression induced by ICH. Furthermore, HE staining and pathological analysis confirmed that rhein intervention alleviated neuronal damage and inflammatory infiltration after ICH.

Notably, NLRP3 knockdown and Caspase‐1 inhibition further enhanced the neuroprotective effects of rhein, effectively reducing inflammatory cytokine expression, pyroptosis‐related protein levels, and GSDMD expression. These findings suggest that ICH‐induced brain injury may be driven by a cascade reaction between NLRP3 inflammasome activation and pyroptosis, contributing to pathological progression, while rhein exerts its protective effects by blocking this critical pathway.

This study demonstrates that rhein alleviates ICH‐induced neuroinflammation and neurological dysfunction by inhibiting NLRP3‐dependent microglial pyroptosis. Importantly, we used a multi‐level experimental design, including in vitro cell models, in vivo collagenase‐induced ICH rats, and mechanistic validation through both NLRP3 knockdown and Caspase‐1 inhibition. Furthermore, we evaluated a broad range of outcomes, from microglial polarization and cytokine release to pyroptotic ultrastructural changes and behavioral recovery. These aspects collectively advance current knowledge by providing robust preclinical evidence that strengthens the translational potential of rhein in hemorrhagic stroke.

Despite its novel findings, this study has certain limitations. First, our in vivo observations were limited to 7 days after ICH induction. This time window was deliberately chosen because spontaneous intracerebral hemorrhage in clinical settings is typically characterized by acute onset and a critical early stage within the first week, during which neuroinflammatory responses and secondary injury are most pronounced. Thus, our study primarily focused on the acute phase of ICH to capture these early pathological processes and therapeutic responses. Nevertheless, we acknowledge that the absence of long‐term behavioral and functional assessments limits our ability to evaluate the sustained efficacy of rhein. Future studies will extend the observation period and include longitudinal assessments to determine whether rhein's neuroprotective effects persist beyond the acute phase. Second, we acknowledge that the use of a single model limits the generalizability of our findings. Incorporating a second model, such as autologous blood injection, would provide external validation and strengthen the translational relevance of our conclusions. Future studies will therefore include multiple ICH models to further substantiate the therapeutic effects of rhein. A further limitation of our study is that the mechanistic exploration was restricted to the NLRP3 inflammasome pathway. Although our findings strongly suggest that rhein exerts protective effects through the inhibition of NLRP3‐mediated pyroptosis, we cannot exclude the involvement of other off‐target or compensatory mechanisms. Comprehensive approaches, including transcriptomic and proteomic profiling, will be necessary in future studies to capture the global molecular responses to rhein and to further substantiate its neuroprotective mechanisms after ICH. In addition, our study did not include formal a priori power calculations to determine sample sizes, which may limit the statistical robustness of the findings. Future studies will incorporate formal sample size calculations to further strengthen the reliability of the results. Finally, this study remains at the preclinical animal research stage. Further preclinical investigations and early‐phase clinical trials are necessary to facilitate the translation of rhein into clinical applications for ICH treatment.

## 5 Conclusion

This study elucidated the mechanism by which rhein improves neurological dysfunction after ICH by inhibiting NLRP3 inflammasome activation, reducing pyroptosis and neuroinflammation levels. Furthermore, it suggests that targeting the NLRP3 pyroptosis pathway could serve as a potential therapeutic strategy for ICH, providing new insights and a theoretical foundation for the clinical treatment of intracerebral hemorrhage.

## Author Contributions

**Adalaiti Aimaiti**: conceptualization, formal analysis, investigation, methodology, writing – original draft. **Qian Li**: conceptualization, formal analysis, investigation, methodology, writing – original draft. **Chen Chen**: Data curation, Investigation, Resources. **Xiaolin Xie**: Project administration, Software. **Jianshu Chu**: Validation. **Dilihumaer Nuermaimaiti**: Validation, Visualization. **Yan Hu**: Software, Visualization. **Tao Liu**: Project administration, writing – review and editing.

## Funding

This study was supported by the Outstanding Youth Science Fund Project of Xinjiang Uygur Autonomous Region (2022D01E79) and Research Innovation Team Project of Xinjiang Medical University (XYD2024C01).

## Ethics Statement

The study was approved by the Animal Ethics Committee of Xinjiang Medical University (No. IACUC‐20210301‐25).

## Conflicts of Interest

The authors declare no conflicts of interest.

## Supporting information




**Figure S1**. Verify the expression of NLRP3 in cells after transfection with shRNA‐NLRP3 adenovirus. (A) The mRNA levels of NLRP3 in cells after transfection with shRNA‐NLRP3 adenovirus. (B) The protein expression of NLRP3 in cells after transfection with shRNA‐NLRP3 adenovirus. (C) The mRNA levels of NLRP3 in cells after transfection with shRNA‐NLRP3 adenovirus. *P < 0.05 compared to Control; # *P* < 0.05 compared to Model.

**Figure S2**. Evaluation of the establishment of ICH models using behavioral studies.

**Figure S3**. Dose‐dependent effects of rhein on behavioral outcomes in ICH rats.

**Figure S4**. Immunofluorescence detection of M1/M2 microglia. Bar = 100 µm.

**Figure S5**. Immunofluorescence detection of M1/M2 microglia. Bar = 100 µm.

**Supplementary Table**: brb371230‐sup‐0006‐Table S1.docx

## Data Availability

The data used to support the findings of this study are available from the corresponding author upon request.
