# Supplementary figures and images for: Rhein Inhibits NLRP3 Activation and Alleviates Microglial Pyroptosis After Intracerebral Hemorrhage in Rats
Source: Brain Behav. 2026 Jan 28;16(2):e71230. doi: 10.1002/brb3.71230 (PMC12848515; doi:10.1002/brb3.71230)

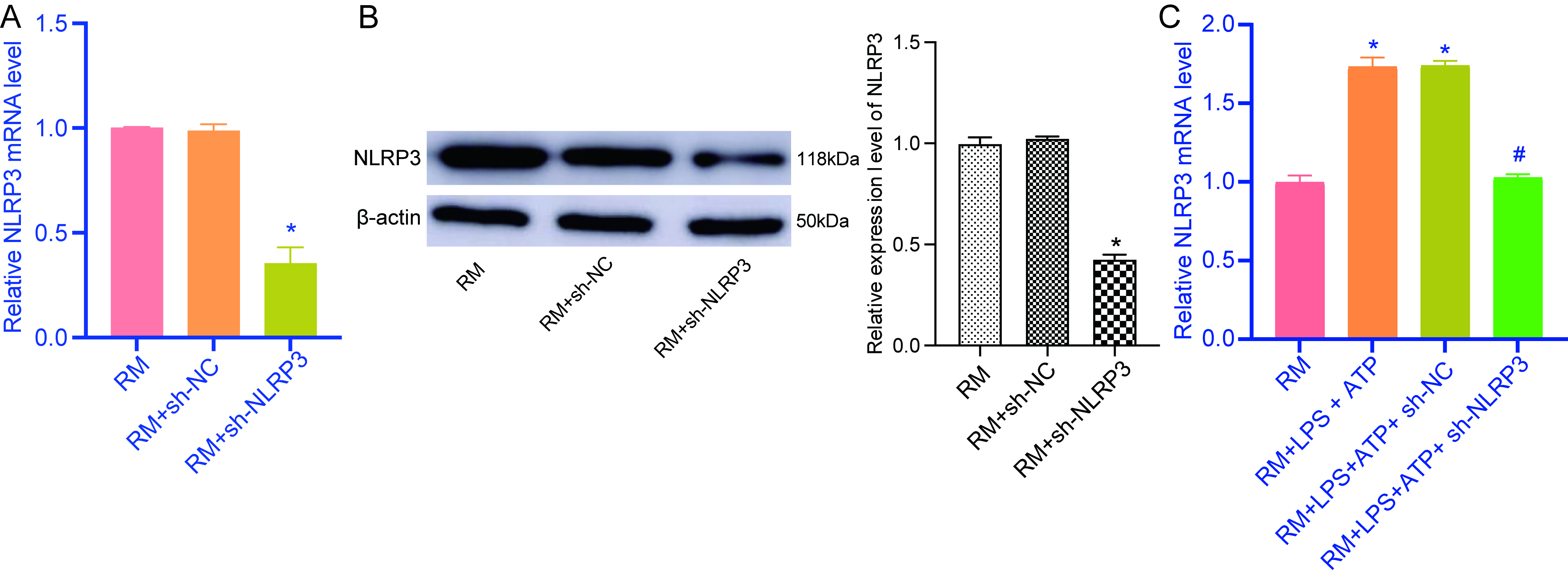

Supplement: Supplementary file 1 — Figure S1. Verify the expression of NLRP3 in cells after transfection with shRNA‐NLRP3 adenovirus. (A) The mRNA levels of NLRP3 in cells after transfection with shRNA‐NLRP3 adenovirus. (B) The protein expression of NLRP3 in cells after transfection with shRNA‐NLRP3 adenovirus. (C) The mRNA levels of NLRP3 in cells after transfection with shRNA‐NLRP3 adenovirus. *P < 0.05 compared to Control; # P < 0.05 compared to Model. [file BRB3-16-e71230-s006.tif]

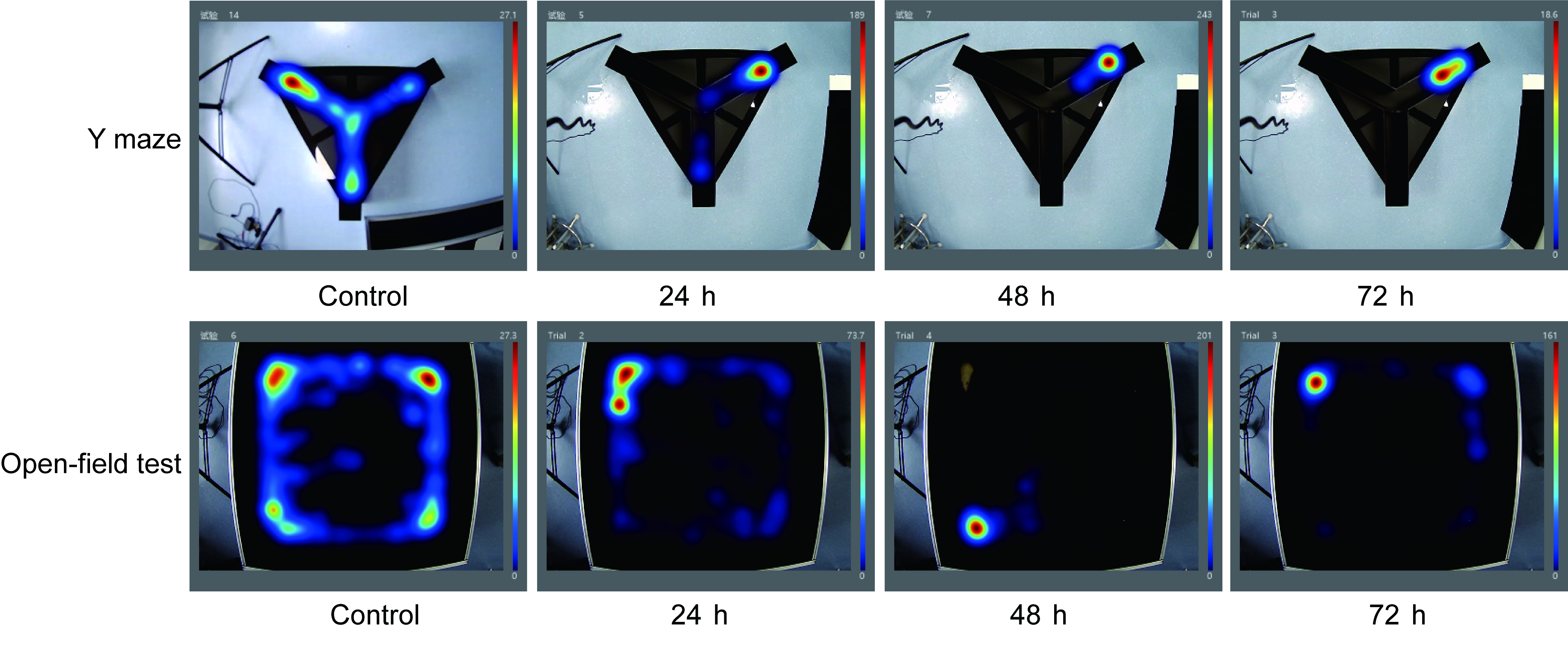

Supplement: Supplementary file 2 — Figure S2. Evaluation of the establishment of ICH models using behavioral studies. [file BRB3-16-e71230-s005.tif]

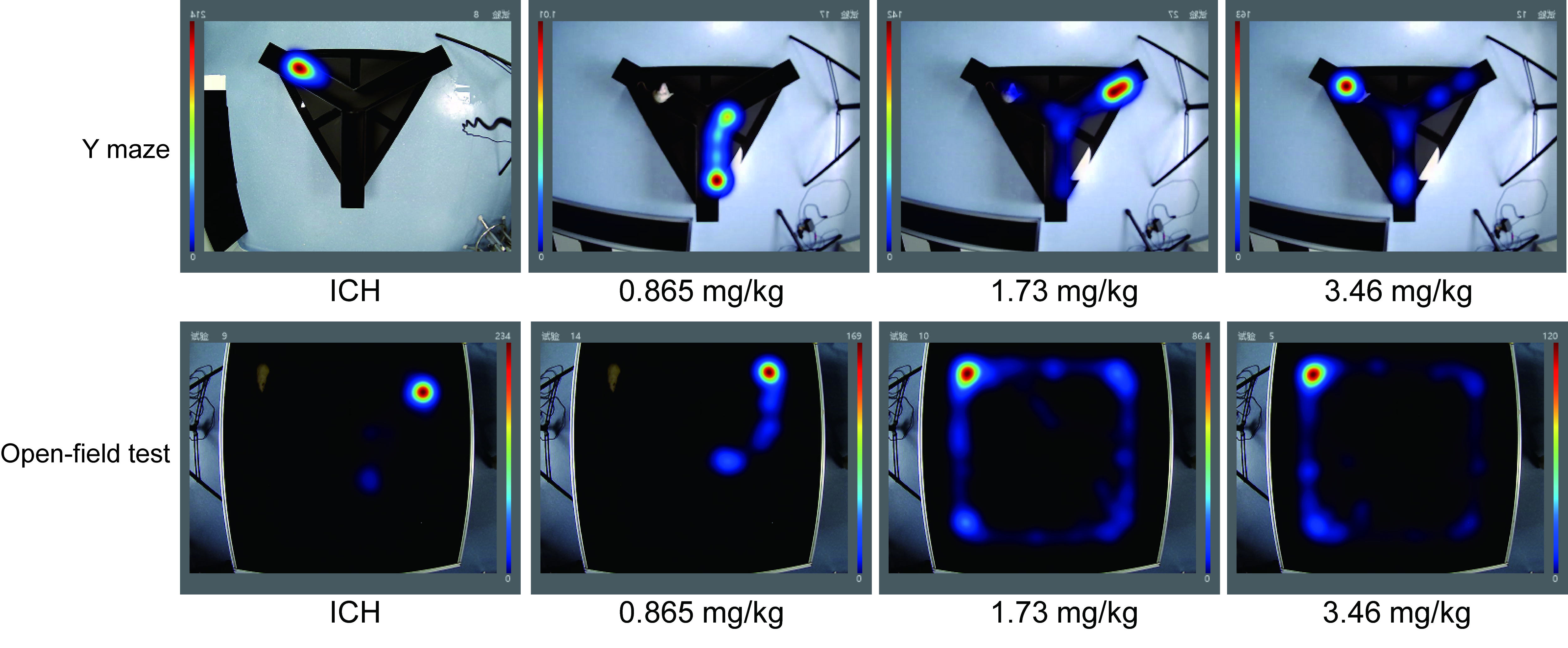

Supplement: Supplementary file 3 — Figure S3. Dose‐dependent effects of rhein on behavioral outcomes in ICH rats. [file BRB3-16-e71230-s003.tif]

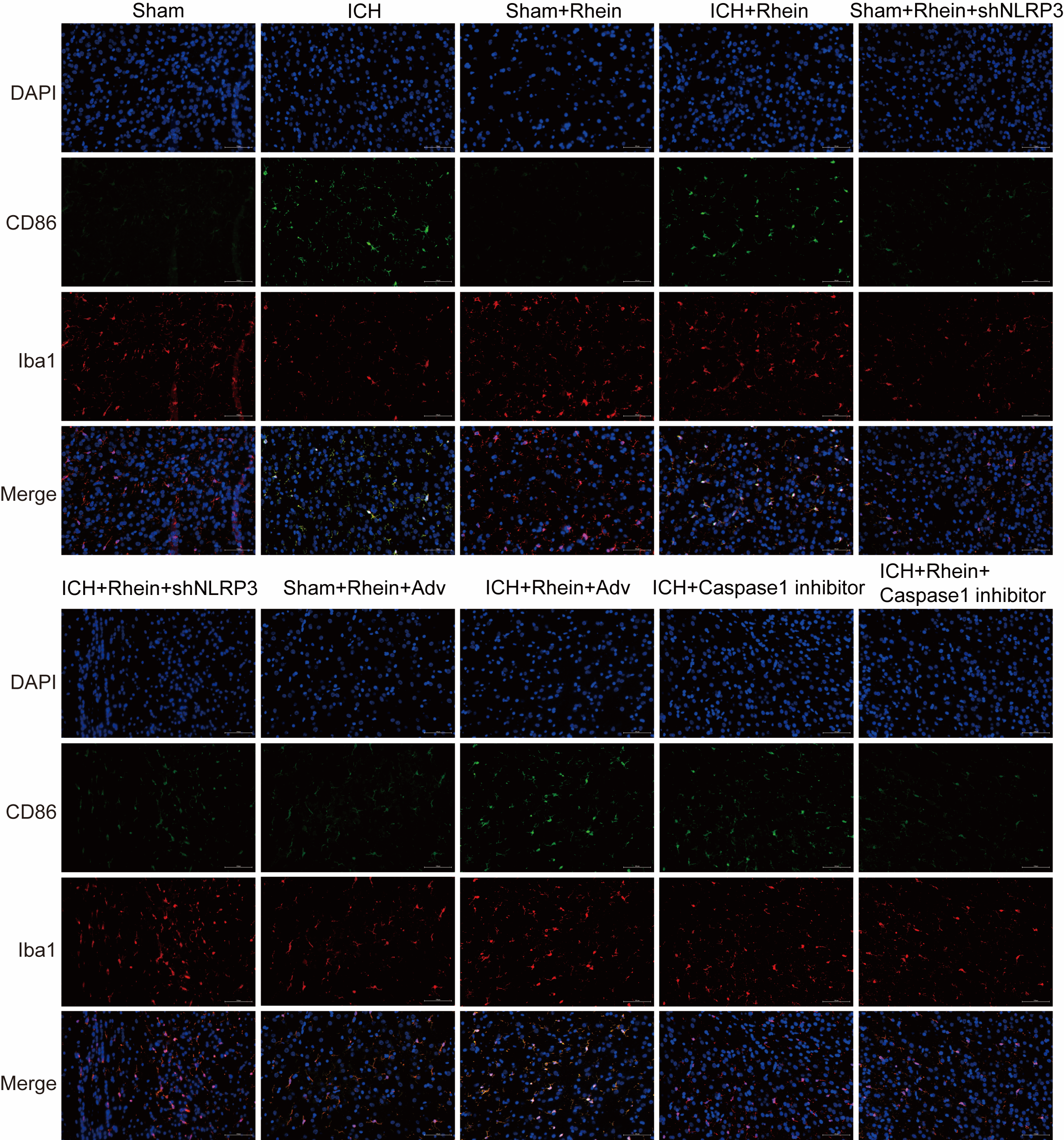

Supplement: Supplementary file 4 — Figure S4. Immunofluorescence detection of M1/M2 microglia. Bar = 100 µm. [file BRB3-16-e71230-s001.tiff]

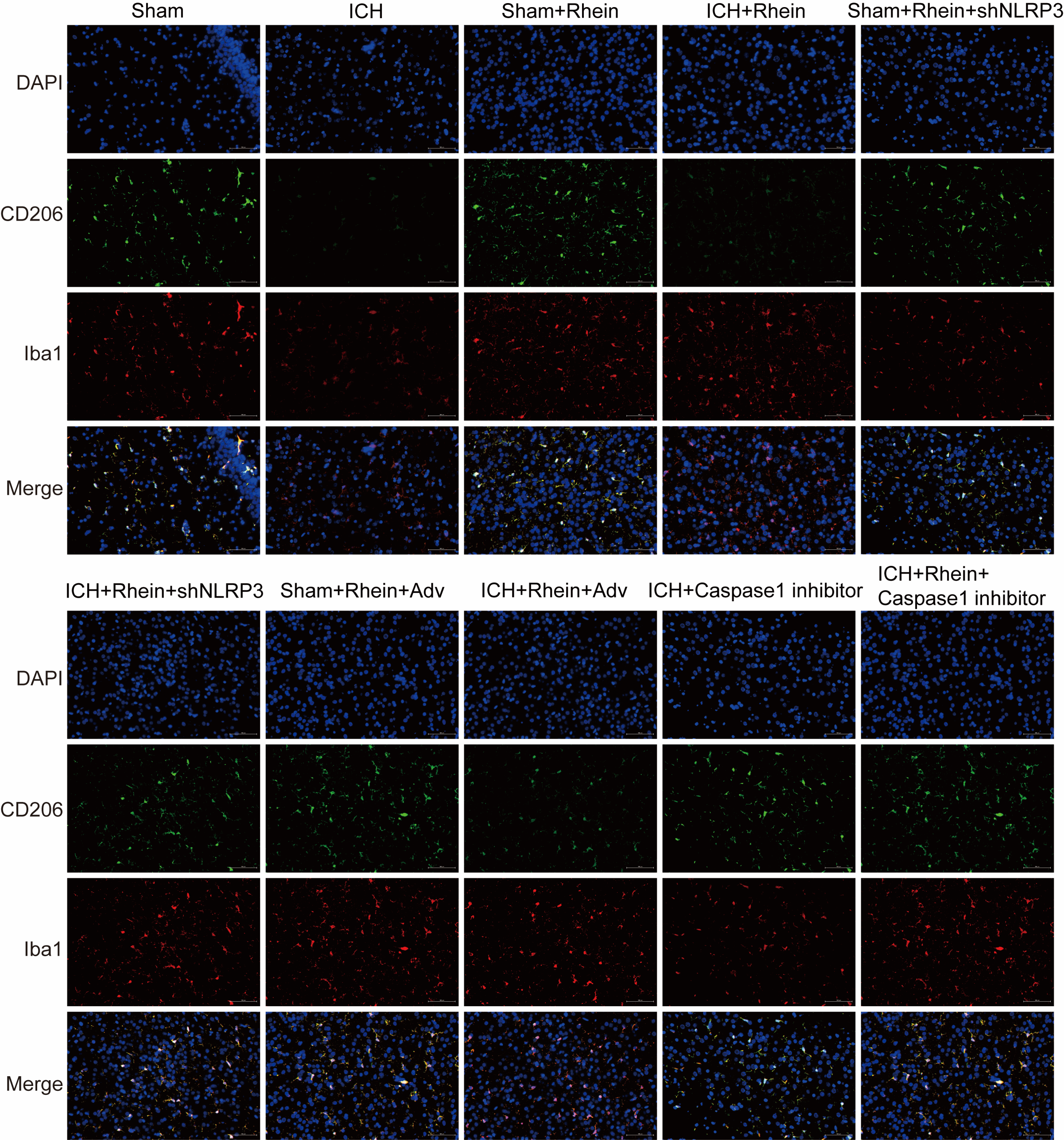

Supplement: Supplementary file 5 — Figure S5. Immunofluorescence detection of M1/M2 microglia. Bar = 100 µm. [file BRB3-16-e71230-s002.tiff]
