# Supplementary material for: Rhein Inhibits NLRP3 Activation and Alleviates Microglial Pyroptosis After Intracerebral Hemorrhage in Rats
Source: Brain Behav. 2026 Jan 28;16(2):e71230. doi: 10.1002/brb3.71230 (PMC12848515; doi:10.1002/brb3.71230)
Supplement: Supplementary file 6 — Supplementary Table: brb371230‐sup‐0006‐Table S1.docx [file BRB3-16-e71230-s004.docx]

Table S1. The primers of this study.

| Genes | Primers (5′-3′) |  |
| --- | --- | --- |
| NLRP3 | F: TGCATGCCGTATCTGGTTGT |  |
|  | R: ATGTCCTGAGCCATGGAAGC |  |
| ASC | F: ACAGTACCAGGCAGTTCGTG |  |
|  | R: GGTCTGTCACCAAGTAGGGC |  |
| Caspase 1 | F: CTCATGGTCTCCAGGAGGGA |  |
|  | R: TCCTTGTTTCTCTCCACGGC |  |
| GSDMD | | F: CTATTCAGCCCTCCCGGAAC |
|  | | R: GCTGTCTGGTATAGTGGGGC |
| PCAN | F: GAACAGGAGTACAGCTGCGT |  |
|  | R: CTCCCCACTCGCAGAAAACT |  |
| Cyclin D1 | | F: TCAAGTGTGACCCGGACTGC |
|  | | R: GGGATCGATGTTCTGCTGGG |
| CDK2 | | F: AGCTCTGCTTGCGTTCCAT |
|  | | R: ACGTGCCCTCTCCAATCTTC |
| β-actin | F: CCCATCTATGAGGGTTACGC |  |
|  | R: TTTAATGTCACGCACGATTTC |  |
